# Case report: Microcirculatory leukocytes in a pediatric patient with severe SARS-CoV-2 pneumonia. Findings of leukocytes trafficking beyond the lungs

**DOI:** 10.3389/fped.2022.978381

**Published:** 2022-09-08

**Authors:** Gabriella Bottari, Can Ince, Valerio Confalone, Salvatore Perdichizzi, Chiara Casamento Tumeo, Joseph Nunziata, Stefania Bernardi, Francesca Calò Carducci, Laura Lancella, Paola Bernaschi, Cristina Russo, Carlo Federico Perno, Corrado Cecchetti, Alberto Villani

**Affiliations:** ^1^Pediatric Intensive Care Unit, Department of Emergency and General Pediatrics, Bambino Gesù Children’s Hospital, IRCCS, Rome, Italy; ^2^Department of Intensive Care, Erasmus MC, University Medical Center Rotterdam, Rotterdam, Netherlands; ^3^Department of Pediatrics, Residency School of Pediatrics, Bambino Gesù Children’s Hospital, IRCCS, University of Rome Tor Vergata, Rome, Italy; ^4^Infectious Diseases and Immuno-Infectiology Unit, Bambino Gesù Children’s Hospital, IRCCS, Rome, Italy; ^5^Unit of Microbiology and Diagnostic Immunology, Department of Diagnostic and Laboratory Medicine, Bambino Gesù Children’s Hospital, IRCCS, Rome, Italy; ^6^Virology and Mycobacteria Unit, Bambino Gesù Children’s Hospital, IRCCS, Rome, Italy; ^7^Multimodal Medicine Research Area, Bambino Gesù Children’s Hospital, IRCCS, Rome, Italy; ^8^Unit of Microbiology and Diagnostic Immunology, Bambino Gesù Children’s Hospital, IRCCS, Rome, Italy; ^9^General Pediatric and Infectious Disease Unit, Department of Emergency and General Pediatrics, Bambino Gesù Children’s Hospital, IRCSS, Rome, Italy

**Keywords:** microcirculation, pediatric critical care, inflammation, micro-thrombosis, SARS-CoV-2, rolling leukocytes

## Abstract

**Background:**

SARS-CoV-2 can lead to excessive coagulation and thrombo-inflammation with deposition of microthrombi and microvascular dysfunction. Several studies in human and animal models have already evidenced biomarkers of endothelial injury during SARS-CoV-2 infection. Real-time observation of sublingual microcirculation using an handheld vital microscopy with an Incident Dark Field (IDF) technique could represent a non-invasive way to assess early signs of microvascular dysfunction and endothelial inflammation in patients with severe COVID-19 infection.

**Clinical case:**

We report for the first time in a pediatric patient with severe SARS-CoV-2 pneumonia findings about microcirculatory leukocytes in the sublingual microcirculation of a 7 month-old patient admitted to our PICU using handheld vital microscopy with IDF technique.

**Results:**

Sublingual microcirculation analysis revealed the presence of microcirculatory alterations and an extensive presence of leukocytes in the patient’s sublingual microcirculation. It’s significant to underline how the patient didn’t show a contextual significant increase in inflammatory biomarkers or other clinical signs related to an inflammatory response, beyond the presence of severe hypoxic respiratory failure.

**Conclusion:**

Leukocyte activation in multiple organs can occur at the endothelial lining of the microvasculature where a surge of pro-inflammatory mediators can result in accumulation of activated leukocytes and degradation of the endothelium. The introduction of a method to assess in a non-invasive, real-time manner the extent of inflammation in a patient with COVID19 could lead to potential clinical and therapeutic implications. However, more studies are required to prove that studying leukocytes microcirculation using sublingual microcirculation analysis could be useful as a bedside point of care monitor to predict the presence of systemic inflammation associated with the impact of COVID-19, leading in a late phase of severe SARS-CoV-2 infection to a microvascular dysfunction and micro-thrombosis.

## Introduction

During infections with highly pathogenic viruses, such as SARS-CoV-2, a failure to rapidly clear these infections can lead to excessive uncontrolled inflammation, resulting in lung injury. However, if SARS-CoV-2 is not efficiently cleared from the respiratory system, in sharp contrast with other pneumotropic viruses, it can spread to other organs using diverse mechanisms of extrapulmonary spread resulting in a variety of clinical manifestations (1). One complication of severe SARS-CoV-2 infection, widely reported in adults and less common in children, is excessive coagulation and thrombo-inflammation, leading to deposition of microthrombi and microvascular dysfunction. Authors have identified endothelial injury in patients with COVID-19, characterized by elevated levels of von Willebrand factor and P selectin, and the presence of activated neutrophils and macrophages in multiple vascular beds, including kidneys, heart, small intestines, and liver (1). Upon binding to the cognate ligand on leukocytes (P-selectin glycoprotein ligand PSGL-1), P-selectin mediates the initial rolling of leukocytes onto the inflamed endothelium which represents the first step in leukocyte recruitment to inflammation sites (2).

Recently, Favaron and co-workers have shown, observing the sublingual microcirculation using handheld vital microscopy with an Incident Dark Field (IDF) technique, a significant enhanced microcirculatory leukocytes number in COVID-19 patients compared to those of healthy volunteers (3). Here is reported for the first time to the best of our knowledge, real-time observations of microcirculatory leukocytes in a 7-month old female patient with severe SARS-CoV-2 pneumonia. Although the authors are aware of the limitations of a single case report, our study highlights the potential role of sublingual microcirculation analysis to monitor not only microvascular dysfunction (3–5), but also the presence of leukocyte recruitment onto the endothelium as the first step of the multisystem thrombo-inflammation observed in severe SARS-CoV-2 infections.

## Case report

In December 2021, a 7 month-old female infant with Down Syndrome, was admitted to another hospital (Annunziata Hospital in Cosenza, Italy) with acute respiratory distress as a result of SARS-CoV-2 infection.

Medical history evidenced that she was born eutocial at 31 weeks of gestation after regular pregnancy. Birth weight 2,750 kg. In last October she was followed at home by the pediatrician for a viral bronchiolitis.

At hospital admission she was first assisted through non-invasive ventilation an underwent a course of antibiotics (Ceftriaxone 50 mg/kg/die), corticosteroids (dexamethasone 0.15 mg/kg/die), and low molecular weight heparin (100 U/kg bpd) according to the local guidelines at that time. As she wasn’t improving clinically, it was also decided to start a 5-day course of Remdesivir (loading dose 5 mg/Kg, then 2.5 mg/kg), two-doses of anti-IL-6 (Tocilizumab) and one administration of monoclonal antibodies (Casirivimab-Imdevimab).

**FIGURE 1:**
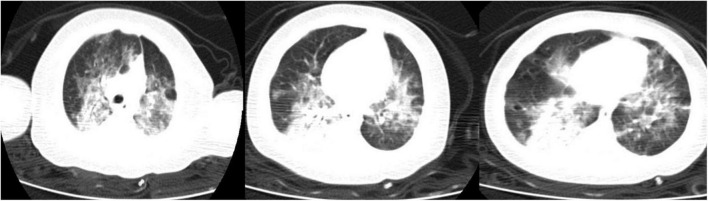
Chest Computed tomography (CT) scan of the patient showing an interstitial pneumonia.

Due to a progressive worsening of the clinical picture she was eventually transferred to the Pediatric Intensive Care Unit of the same hospital. The chest-CT scan, performed on the same day her clinical conditions worsened, showed evidence of interstitial pneumonia evolving to an irregular paving pattern (Figure 1). Unfortunately, she further worsened developing a hypoxic respiratory failure for which invasive mechanical ventilation with an FiO2 of 100% was started.

On day 15th of her hospitalization, it was decided, due to the lack of clinical improvement, to transfer the patient to our PICU for potential need of extra-corporeal membrane oxygenation (ECMO).

Once she was admitted to our hospital, SARS-CoV-2 infection was confirmed through real-time polymerase chain reaction (RT-PCR) on a nasopharyngeal swab and bronco-alveolar lavage (BAL). The microbiological work-up evidenced a positive blood culture for multisensitive “Candida albicans.” Blood chemistry gave the following values: White Blood Cells (WBC) 13.0 10^3^/μL, Hemoglobin 15 gr/dl, Platelets 137 10^3^/μL, Neutrophils 10.7 10^3^/μL Lymphocytes 1.59 10^3^/μL, C-Reactive Protein and Procalcitonin returned negative, Ferritin 552 ng/ml, D-dimers 1.96 μg/ml, Anti-thrombin III 104%, hepatic and renal biomarkers were in the normal range.

At the time of admission, the patient was hemodynamically stable without need of inotropes or vasopressors (lactate min 1.2–max 2.9 mmol/L). She continued invasive mechanical ventilation (Peep 10 Pip 24 RR 24 FiO2 100%) in association with nitric oxide 20 ppm and prone-supine position. Arterial gas analysis showed a PaO2/FiO2 ratio min 95–max 160 mmHg; paO2 min 76–max 100 mmHg, pCO2 min 38–max 44 mmHg).

We continued ceftriaxone (50 mg/kg/day), dexamethasone (0.15 mg/kg/day), low molecular weight heparin (100 U/kg bpd) and, in order to treat “Candida albicans,” Micafungine was added.

Due to the child’s critical conditions, it was also decided to prolong the therapy with Remdesivir to 10 days in total (2.5 mg/kg/die) and she also received intravenous immunoglobulins (400 mg/kg/die) for 5 days.

At admission, sublingual microcirculation measurements using handheld vital—microscopy with an IDF technology (Cytocam, Braedius Medical, Huizen, Netherlands) were performed (Supplemental https://doi.org/10.6084/m9.figshare.20463483.v3). Briefly, the IDF imaging technique uses green light produced from a ring of circumferential light-emitting diodes at the end of a light guide through a magnifying lens. The green light is transmitted to the tissue and absorbed by Hb; thus, the RBCs appear as dark globules and WBCs as white globules. The imaging results in sharp contour visualization of the microcirculation, showing flowing RBCs and leukocytes captured by a high-definition image sensor. The IDF handheld vital microscope captures images at a rate of 25 frames/s, of which 100 frames are recorded in a single video clip (6). ***Image acquisition:*** Sublingual video clips with at least one postcapillary venule unit (C-PCV) were included for analysis. The selection criterion used to define a C-PCV vessel segment was that a Capillary (Cap) should merge into a PCV (small venule distal to the capillary) with no branching vessels present (7). Study of the C-PCV unit enables the tracking of WBCs from the Cap into the PCV thereby identifying the presence of activated leukocytes. ***Leukocytes identification:*** We made a quantitative assessment of leukocytes using a manual counting methodology in sublingual recordings subdividing the screen into 9 rectangles. We counted the number of C-PCV in each square and the number of leukocytes that were identified in each C-PCV (8). Furthermore, to distinguish between leukocytes and plasma gaps (PGs) and to identify the nature of the kinetics of the leukocytes in the capillaries and venules, we analyzed the microcirculatory images using space time diagrams (STD) (7). STDs of single vessels can be generated using specialized softwares such as the Analysis Manager V2 software, which was originally developed for the measurement of RBCs velocity and requires user interactions. Briefly, the y-axis of the STD corresponds to the length of the detected vessel segment. The x-axis corresponds to time in line with the frame number. Each column represents the image intensity along a vessel centerline of one video frame. Alternating black and white bands in the STDs are formed by flowing RBCs and white bands of PGs or leukocytes, respectively. The slopes of the black and white bands in these diagrams represent the velocity (distance/time velocity), RBCs accumulation behind the larger and slower leukocytes is defined as the “train formation” of RBCs and is detectable in the STDs as a large dark band next to the large white band. These dark bands behind the leukocytes caused by the train formation of the RBCs are larger than the black lines of the RBCs and the white lines of the PGs. Leukocytes were further subdivided into non-rolling (NRL) and rolling cells (RL). Rolling leukocytes were identified by the marked deviation in the titled bands on the STDs. The change in the slope marked slowdown in the leukocyte velocity. Non-rolling leukocytes showed a linear (straight) within the band on the STD (7).

**FIGURE 2:**
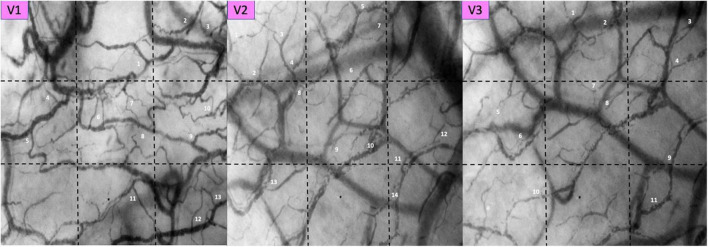
Pictures of each selected video. Post Capillaries Venules (PCV) units are identified by sequential numbers and associated to the space-time diagrams analyses in Figure 3.

**FIGURE 3:**
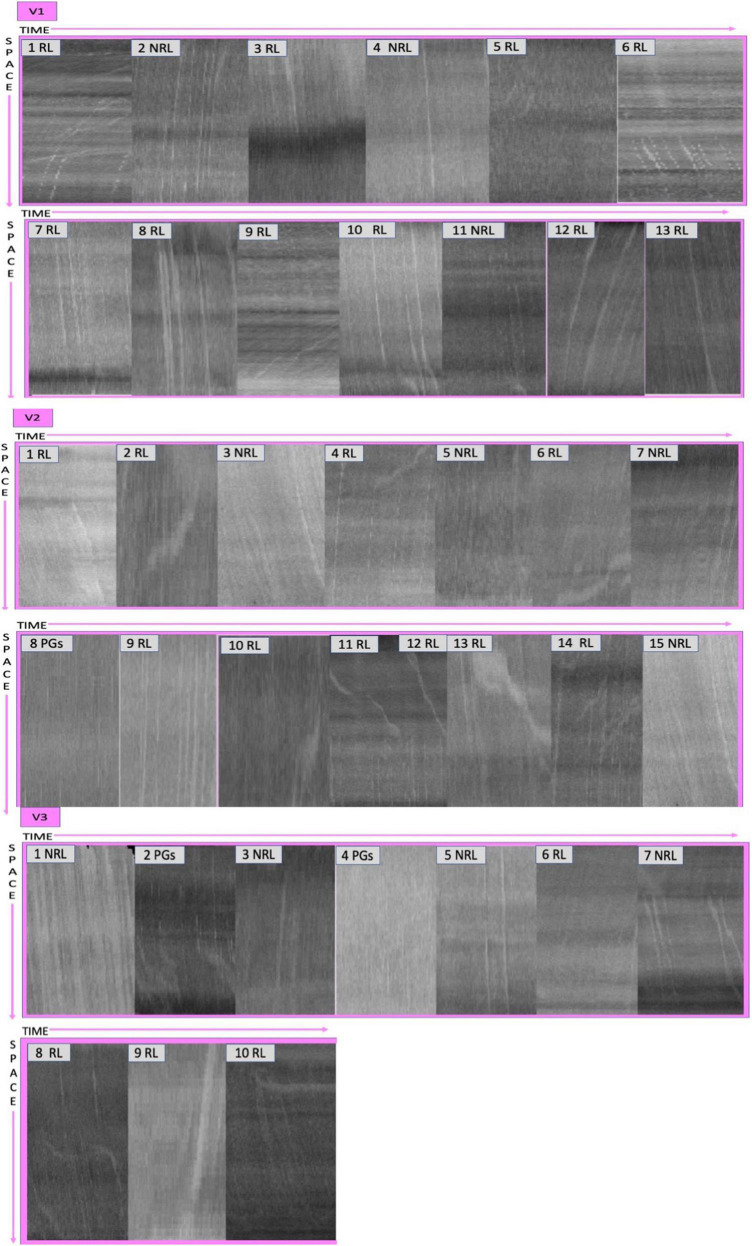
Space time diagrams describing the behavior of the leukocytes in PCV-units. RL, rolling leukocytes; NRL, no-rolling leukocytes; PGs, plasma gaps.

Sublingual microcirculation analysis revealed not only the presence of microcirculatory alterations characterized by RBCs with sluggish and no-flow vessels, as already reported in our studies (4), but also an extensive presence of microcirculatory leukocytes. In Table 1 we present the numbers of PCV units per selected each video, in Figures 2, 3 the pictures of each selected video associated to the space-time diagram for each PCV-unit and the respective space time diagram describing the behavior of the leukocytes (rolling vs. non-rolling).

**TABLE 1:** Table shows numbers of post capillaries venules (PCV) units in each video selected (total) and absolute numbers of rolling leukocytes and non-rolling leukocytes in PCV-units observed counted during 100 frames videos.

**Video 1**	
P-CV units (total)	13
n° P-CV units rolling leukocytes	10
n° P-CV units non-rolling leukocytes	3
**Video 2**	
P-CV units (total)	14
n° P-CV units rolling leukocytes	9
n° P-CV units non-rolling leukocytes	5
**Video 3**	
P-CV units (total)	10
n° PCV units rolling leukocytes	4
n° PCV units non-rolling leukocytes	5

On day 23rd of her hospitalization, the patient developed a septic shock (9) which needed norepinephrine (max dose required 0.15 μg/kg/min) and antimicrobial therapies were implemented with piperacillin-tazobactam (100 mg/kg tpd), meropenem (40 mg/kg tpd) and amphotericin B (5 mg/kg). Dexamethasone was reduced for a persistent leukopenia and central venous access was changed. Blood chemistry on this day showed a WBC count of 4.26 10^3^/μL, Hemoglobin 9.8 gr/dl, Platelets 50 10^3^/μL, Neutrophils 2.62 10^3^/μL Lymphocytes 1.14 10^3^/μL, C-Reactive Protein 2.48 mg/dL; Procalcitonin 2.58 ng/ml; Ferritin 1421 ng/ml, D-dimers 4.61 μg/ml, Anti-thrombin III 88%, hepatic and renal biomarkers remained in normal range limits.

**FIGURE 4:**
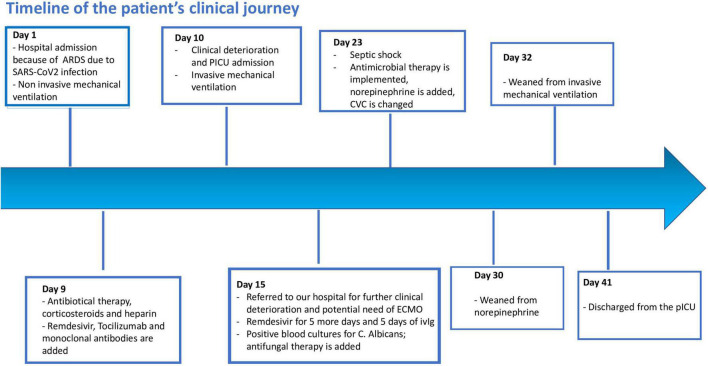
Patient clinical journey showed by a graphical timeline.

A week later she progressively improved and norepinephrine was stopped. She was weaned by invasive mechanical ventilation on day 30th and discharged by PICU on day 41st (Figure 4).

## Discussion

Leukocyte activation in multiple organs can occur at the endothelial lining of the microvasculature where a surge of pro-inflammatory mediators can result in the accumulation of activated leukocytes and degradation of the endothelium. Studies have shown that degradation of the endothelium results in capillary leakage and disruption in capillaries. The assessment of leukocytes—endothelium interactions within the microcirculation has been made possible through the introduction of handheld vital microscopy and the sublingual area is the optimal target for monitoring critically ill patients in a non-invasive manner (10).

SARS-CoV-2 infection is considered to be highly diffuse beyond the lung with known lethal effects related to the thrombo-inflammation culminating in multiple organ damage. Already other authors have shown the impact of endothelial damage in critically ill children with COVID-19 severe infection and Multisystem Inflammatory Syndrome (MIS-C) SARS-CoV-2 related using non-invasive IDF or sidestream dark field (Glycocheck System) to perform *in vivo* identification of microvascular dysfunction and glycocalyx damage (4, 11, 12): in the present study is reported for the first time to the best of our knowledge in a critical ill child affected by severe SARS-CoV-2 pneumonia findings related to microcirculatory leukocytes. It’s significant to underline how our patient did not show a significant increase in inflammatory biomarkers or other clinical signs related to a systemic inflammatory response, beyond the presence of severe hypoxic respiratory failure which required mechanical ventilation. Despite this lack of clinical symptoms related to inflammation, we observed, using sublingual microcirculation observation, the presence of an extensive number of leukocytes and their activation with a high percentage of rolling leukocytes vs. non-rolling leukocytes. On the other hand as an important limit of our paper it emerges that the child at PICU admission showed since the first day positive blood cultures for “Candida albicans” thus, however, the first hypothesis is a central line colonization an impact on endothelial inflammation by this element cannot be fully excluded.

The present sublingual measurements allowed us to observe real time, in a severe SARS-CoV-2 pneumonia, the cellular impact of systemic inflammation induced by COVID-19 infection, justifying our therapeutic approach. However, more studies are required to provide further evidences that studying leukocytes microcirculation with bedside techniques could be useful point of care monitor to predict the presence of systemic inflammation associated with COVID-19, leading in a late phase of severe SARS-CoV-2 infection to microvascular dysfunction and micro-thrombosis.

## Conclusion

Sublingual microcirculation analysis performed with the IFD technique allowed us to identify enhanced leukocyte trafficking beyond the lungs in a pediatric clinical case of severe SARS-CoV-2 pneumonia, despite normal biomarkers of inflammation. Our report not only confirms earlier findings in adults, but also demonstrates that neutrophils recruitment can be non-invasively and safely monitored in critically ill children with SARS-CoV-2 infection using bedside microcirculatory monitoring to predict the consequences of thrombo-inflammation related to the virus.

## Data availability statement

The original contributions presented in this study are included in the article/supplementary material, further inquiries can be directed to the corresponding author.

## Ethics statement

The studies involving human participants were reviewed and approved by the IRCCS Bambino Gesù Children’s Hospital. Written informed consent to participate in this study was provided by the participants’ legal guardian/next of kin. Written informed consent was obtained from the minor(s)’ legal guardian/next of kin for the publication of any potentially identifiable images or data included in this article.

## Author contributions

GB and CI drafted the manuscript. All authors were involved in the clinical management, analyzed and interpreted the data, discussed the results, read, and approved the final manuscript.

## References

[1] Alon R Sportiello M Kozlovski S Kumar A Reilly EC Zarbock A Leukocyte trafficking to the lungs and beyond: lessons from influenza for COVID-19. *Nat Rev Immunol.* (2021) 21:49–64. 10.1038/s41577-020-00470-2 33214719 PMC7675406

[2] Neri T Neri D Celi A. P-selectin blockade in COVID-19-related ARDS. *Am J Physiol Lung Cell Mol Physiol.* (2020) 318:L1237–8. 10.1152/ajplung.00202.2020 32464083 PMC7276981

[3] Favaron E Ince C Hilty MP Ergin B van der Zee P Uz Z Capillary leukocytes, microaggregates, and the response to hypoxemia in the microcirculation of coronavirus disease 2019 patients. *Crit Care Med.* (2021) 49:661–70. 10.1097/CCM.0000000000004862 33405410 PMC7963442

[4] Damiani E Carsetti A Casarotta E Scorcella C Domizi R Adrario R Microvascular alterations in patients with SARS-CoV-2 severe pneumonia. *Ann Intensive Care.* (2020) 10:60. 10.1186/s13613-020-00680-w 32436075 PMC7238400

[5] Aykut G Veenstra G Scorcella C Ince C Boerma C. Cytocam-IDF (incident dark field illumination) imaging for bedside monitoring of the microcirculation. *Intensive Care Med Exp.* (2015) 3:40. 10.1186/s40635-015-0040-7 26215807 PMC4512989

[6] Uz Z van Gulik TM Aydemirli MD Guerci P Ince Y Cuppen D Identification and quantification of human microcirculatory leukocytes using handheld video microscopes at the bedside. *J Appl Physiol.* (2018) 124:1550–7. 10.1152/japplphysiol.00962.2017 29517420

[7] Bauer A Kofler S Thiel M Eifert S Christ F. Monitoring of the sublingual microcirculation in cardiac surgery using orthogonal polarization spectral imaging: preliminary results. *Anesthesiology.* (2017) 107:939–45. 10.1097/01.anes.0000291442.69337.c9 18043062

[8] Goldstein B Giroir B Randolph A. “International pediatric sepsis consensus conference: definition for sepsis and organ dysfunction in pediatrics”. *Pediatr Crit Care.* (2006) 6:2–8. 10.1097/01.PCC.0000149131.72248.E6 15636651

[9] Uz Z Ince C Shen L Ergin B van Gulik TM. Realt-time observation of microcirculatory leukocytes in patients undergoing major liver resection. *Sci Rep.* (2021) 11:4563. 10.1038/s41598-021-83677-0 33633168 PMC7907405

[10] Fernandez Sarmiento J Florez S Alarcon-Forero LC Salazar-Pelaez LM Garcia-Casallas J Mulett H Case report: endothelial glycocalyx damage in critically ill patients with SARS-CoV2- related multisystem inflammatory syndrome (MIS-C). *Front Pediatr.* (2021) 9:726949. 10.3389/fped.2021.726949 34552899 PMC8451682

[11] Bottari G Confalone V Cotugno N Guzzo I Perdichizzi S Manno EC Efficacy of cytosorb in a pediatric case of severe multisystem inflammatory syndrome (MIS-C): a clnical case report. *Front Pediatr.* (2021) 11:676298. 10.3389/fped.2021.676298 34178891 PMC8232055

[12] Bottari G Damiani E Confalone V Scorcella C Casarotta E Gandolfo C Microvascular dysfunction in pediatric patients with SARS-CoV-2 pneumonia: report of three severe cases. *Microvasc Res.* (2022) 141:104312. 10.1016/j.mvr.2022.104312 35026289 PMC8744301

